# Atlas-Free Cervical Spinal Cord Segmentation on Midsagittal T2-Weighted Magnetic Resonance Images

**DOI:** 10.1155/2017/8691505

**Published:** 2017-05-04

**Authors:** Chun-Chih Liao, Hsien-Wei Ting, Furen Xiao

**Affiliations:** ^1^Institute of Biomedical Engineering, National Taiwan University, No. 1, Sec. 1, Renai Rd., Taipei City 10051, Taiwan; ^2^Department of Neurosurgery, Taipei Hospital, Ministry of Health and Welfare, No. 127, Siyuan Rd., New Taipei City 24213, Taiwan; ^3^Department of Information Management, Yuan Ze University, No. 135, Yuan-Tung Road, Chungli, Taoyuan 32003, Taiwan; ^4^Department of Neurosurgery, National Taiwan University Hospital, No. 7, Zhongshan S. Rd., Taipei City 10002, Taiwan

## Abstract

An automatic atlas-free method for segmenting the cervical spinal cord on midsagittal T2-weighted magnetic resonance images (MRI) is presented. Pertinent anatomical knowledge is transformed into constraints employed at different stages of the algorithm. After picking up the midsagittal image, the spinal cord is detected using expectation maximization and dynamic programming (DP). Using DP, the anterior and posterior edges of the spinal canal and the vertebral column are detected. The vertebral bodies and the intervertebral disks are then segmented using region growing. Then, the anterior and posterior edges of the spinal cord are detected using median filtering followed by DP. We applied this method to 79 noncontrast MRI studies over a 3-month period. The spinal cords were detected in all cases, and the vertebral bodies were successfully labeled in 67 (85%) of them. Our algorithm had very good performance. Compared to manual segmentation results, the Jaccard indices ranged from 0.937 to 1, with a mean of 0.980 ± 0.014. The Hausdorff distances between the automatically detected and manually delineated anterior and posterior spinal cord edges were both 1.0 ± 0.5 mm. Used alone or in combination, our method lays a foundation for computer-aided diagnosis of spinal diseases, particularly cervical spondylotic myelopathy.

## 1. Introduction

The human spinal cord is a long cylindrical structure of the central nervous system extending from the medulla oblongata. Its function is relaying neural signals between the brain and the rest of the body. Residing within the spinal canal formed by the spinal vertebrae, the spinal cord is prone to external compression caused by degeneration, trauma, and so forth. Pathological conditions affecting the spinal cord, also known as myelopathy, lead to motor, sensory, and autonomic dysfunctions, as well as a reduction in quality of life [1]. Among them, cervical spondylotic myelopathy (CSM) is the commonest cause of spinal cord dysfunction in adults globally [2].

Current radiological modality of choice to assess the severity of cervical myelopathy is magnetic resonance imaging (MRI). It provides information about the etiology of canal stenosis, the degree of cord compression, and pathological changes within the cord [3]. Fehlings et al. measured canal compromise on computed tomographic (CT) and T1- and T2-weighted MR images, as well as cord compression on T1- and T2-weighted MR images from patients with spinal cord injury [4]. Based on these methods, experts have developed standardized measurements on midsagittal MR images to quantitatively assess the severity of cord compression in cervical myelopathy in recent years [1–3]. Automation of these measurements requires segmentation of the spinal cord, whether compressed or not, in the original MR images. To our knowledge, no such attempt has been reported.

Current automatic or semiautomatic spinal cord segmentation algorithms focus on multiple sclerosis, which causes atrophy manifested as a decreasing spinal cord area in MR images [5–8]. The earliest semiautomatic method based on an active surface overestimated the cord area in T1-weighted images by approximately 14%, compared to manual outlining [5]. To initialize the algorithm, a human user must mark the approximate cord centerline on a few representative slices. Deformable atlas and Hough transform were employed in newer methods to decrease human intervention used to detect the cord in axial images as well as to improve segmentation accuracy [6, 7]. The Dice coefficients were around 0.9 for the T1-, T2-, and T2∗-weighted images. For spinal cord segmentation in MR images from patients with CSM, these methods may encounter a problem when the cerebrospinal fluid (CSF) spaces outside the cord are compressed secondary to canal stenosis, reducing local tissue contrast.

In the literature, there were only a few atlas-free segmentation methods for the human spinal canal or spinal cord [9, 10]. Archip et al. presented a knowledge-based approach to identify the spinal cord in CT images of the thorax [9]. They constructed a task-oriented anatomical structure map to define the lumbar vertebrae. Although they employed knowledge at incorrect body regions, the results were useable because bony structures are the brightest ones and have fairly stable intensity levels. Kawahara et al. proposed a method to find the globally optimal segmentation of the spinal cord using a high dimensional minimal path search [10]. They represent spinal cord shape principal component analysis. Then, a modified *A*∗ minimal path search algorithm in six dimensions was used. Despite dramatically reduced memory requirement, their run-time was between 1 and 5 hours per case.

In this paper, we report an automatic atlas-free algorithm that can perform cervical spinal cord segmentation in standard T2-weighted sagittal MR images without any preprocessing. Human intervention is minimized. Without an atlas, the anatomical knowledge is transformed into constraints employed at different stages of the algorithm. Our method is able to find the spinal cord in images from patients without disruption of the spinal canal. We applied this method to a large number of consecutive patients undergoing a noncontrast MRI study over a 3-month period. The results are presented and evaluated.

## 2. Materials and Methods

### 2.1. Materials

All adult subjects undergoing noncontrast cervical spine MRI examination from October to December 2015, mainly for CSM, at a regional hospital in Northern Taiwan were retrospectively identified in the database. Patients with a history of cervical spine surgery were excluded. Sagittal T2-weighted images from the subjects were downloaded from the picture archiving and communication system to a personal computer in lossless JPEG format. Our data collection process conformed to the requirements of Institutional Review Board, Taipei Hospital, Department of Health, Taiwan, and was approved as such (TH-IRB-0016-0001).

Image acquisitions were performed on a Siemens Magnetom Avanto 1.5 Tesla MRI scanner (Siemens Healthcare, Erlangen, Germany) using standard coils. Each subject had a T1- and a T2-weighted scan covering the full cervical spinal cord. Parameters for the T2-weighted scan were turbo spin echo sequence, TR = 3300 ms and TE = 95 ms; flip angle = 150°; bandwidth = 223 Hz/voxel; number of averages = 2; and reconstruction diameter = 22 × 22 cm. For sagittal T2 sequence, 3 mm sagittal slices with 0.33 mm gaps between them were planned over the coronal image to cover the whole spinal canal [11]. A saturation band is placed over the anterior, inferior aspect. A total of 13 gray scale images were generated in each sagittal T2 scan. These images are 320 × 320 pixels in size, with a resolution of 0.6875 mm per pixel. The signal intensities (SIs) of the pixels assume a relative scale, stored in 256 gray levels.

### 2.2. Symmetry-Based Selection of the Midsagittal Image

**Figure 1:**
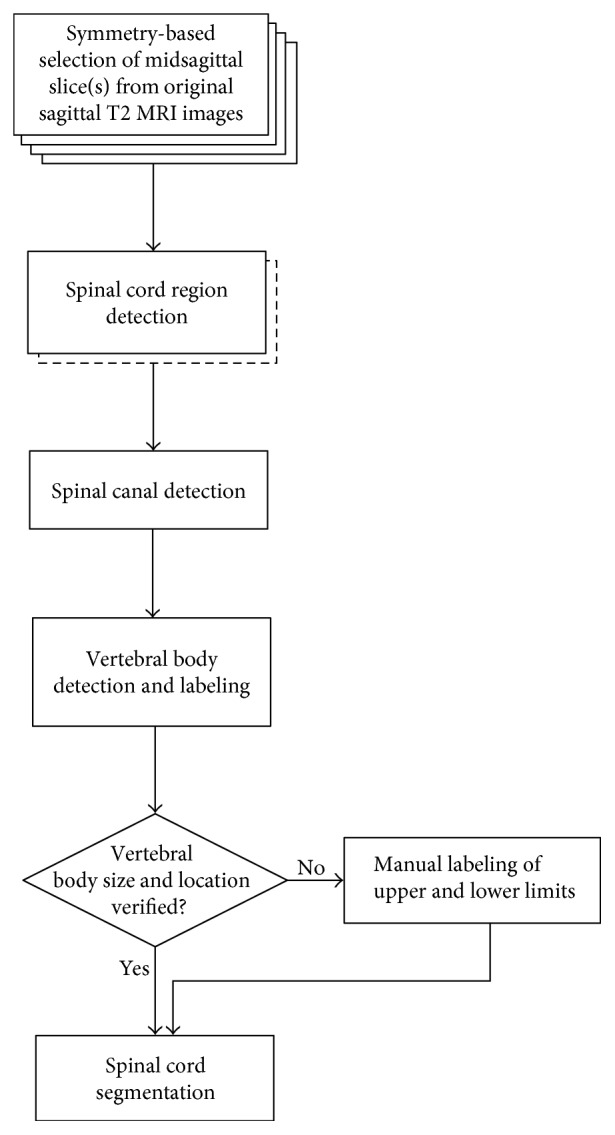
The flowchart of our algorithm.

**Figure 2:**
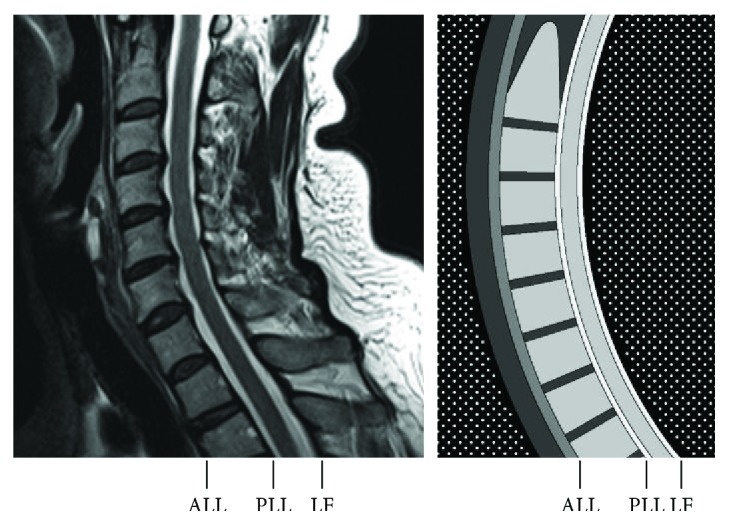
A midsagittal T2-weighted MR image (left) and our schematic drawing showing the spinal cord and its surrounding structures (right). The ligaments, including the anterior longitudinal ligament (ALL), the posterior longitudinal ligament (PLL), and the ligamentum flavum (LF), are deliberately thinned, and the internal architectures of intervertebral disks are neglected.

The flowchart of our algorithm is shown in Figure 1. For each MRI data set, we start from selection of one or two midsagittal images based on symmetry between pairs of images. The spinal cord is detected using expectation maximization (EM) and dynamic programming (DP). Only one image is designated as the midsagittal image and undergoes further processing according to the model depicted in Figure 2.

Using DP, the anterior and posterior edges of the spinal canal are detected, as well as the approximate anterior edge of the vertebral bodies (VBs). After thresholding and DP, the VBs and the intervertebral disks were segmented using region growing and then labeled according to their relative sizes. The superior and inferior edges of the cervical VBs were verified by the user and corrected as needed. Finally, the spinal cord is segmented by using DP to detect its anterior and posterior edges.

We define *x*-, *y*-, and *z*-axes as left-right, anterior-posterior (ventral-dorsal), and superior-inferior (cranial-caudal) axes, respectively. Measurements performed on *x*-, *y*-, and *z*-axes are termed width, depth, and height unless otherwise specified. *xy*, *yz*, and *xz* planes correspond to axial, sagittal, and coronal anatomical planes. A sagittal T2-weighted scan contains 13 gray scale images, denoted as *I* ^*k*^, where *k* = 1, 2,…, 13. Let *I*_*y*,*z*_^*k*^ denote the gray level of the pixel at position (*y*, *z*) of the *k*th sagittal image *I* ^*k*^, 0 ≤ *I*_*y*,*z*_^*k*^ ≤ 255 for 1 ≤ *y*, *z* ≤ 320.

Similar to the brain, the human vertebral column and the spinal cord are bilaterally symmetric about the intact midsagittal plane (iMSP) [12]. We apply this knowledge to identify the midsagittal MR image, which usually contains the largest anterior-posterior (A-P) diameter of the cervical spinal cord. Let *k*_MSP_ denote the sagittal position closest to the iMSP. We have to define a difference metric between two images *I* ^*p*^ and *I* ^*q*^, denoted as *D*(*I* ^*p*^,  *I* ^*q*^), and then test different trial values of *k*_MSP_ to find the best one minimizing the global asymmetry quantified using several pairs of corresponding images.

(1) $$\begin{array}{c} k_{MSP}=\underset{k}{arg\;min}\left(\overset{13}{\underset{j=1}{\sum }}\frac{D\left(I^{j},I^{2k-j}\right)}{m}\right), \end{array}$$

where *m* denotes the number of image pairs.

Theoretically, *k*_MSP_ can assume any real value between 1 and 13 in our images, with an ideal value of 7 for a perfectly positioned patient. However, interpolating images take additional time and generate noisy in-between images, so we only use original images to evaluate global symmetry. Since *D*(*I* ^*p*^, *I* ^*q*^) is defined only when *p* and *q* are integers, *k*_MSP_ must be an integer or a half-integer. When *k*_MSP_ is an integer, *I* ^*k*_MSP_^ is the only image near the iMSP. When it is a half-integer, *I* ^*k*_MSP_−0.5^ and *I* ^*k*_MSP_+0.5^ are the two nearest images located on both sides of the iMSP, but they are usually not equally distant from it. To make meaningful comparison using enough number of images, we only evaluate candidate values when there are at least 4 image pairs available; thus, 4 ≤ *k*_MSP_ ≤ 10.

Several functions can be chosen as the definition of *D*(*I* ^*p*^, *I* ^*q*^), including the mean or standard deviation of the SI difference, cross-correlation between corresponding pixels, and the negative of mutual information (MI) using joint histogram. After a pilot study, we found that the standard deviation of the corresponding pixels' gray level differences between *I* ^*p*^ and *I* ^*q*^ performs best empirically, so *D*(*I* ^*p*^, *I* ^*q*^) is defined as such. As a result, *D*(*I* ^*p*^, *I* ^*q*^) = *D*(*I* ^*q*^, *I* ^*p*^) and *D*(*I* ^*p*^, *I* ^*p*^) = 0 for the same image. After computing *D*(*I* ^*p*^, *I* ^*q*^) for all 78 image pairs, the *k*_MSP_ of the given data set can be found.

### 2.3. Spinal Cord Detection Using Expectation Maximization and Dynamic Programming

Table 1 lists SIs of different tissues on T2-weighted MR images. The spinal cord generally has a smooth contour throughout its course, as shown in Figure 2. It is isointense to the brainstem on all imaging sequences, while the surrounding CSF demonstrates characteristic hyperintensity on T2-weighted images [13]. On images, the spinal cord serves as the reference to classify pixels into hyper-, iso-, and hypointense ones, which have SIs higher than, similar to, and lower than it, respectively.

The normal appearance of the VBs is determined by the ratio of fatty yellow marrow to the hematopoietic red marrow, while their bony cortex demonstrates low SI. The three major components of an intervertebral disk include the nucleus pulposus, annulus fibrosus, and cartilaginous end plate. Only the nucleus pulposus in its core demonstrates high SI due to high water content, which decreases with age. The other two structures demonstrate low SI and are difficult to differentiate from the surrounding vertebral cortex and ligaments.

The ligaments of the spine include the anterior longitudinal ligament (ALL), the posterior longitudinal ligament (PLL), and the posterior ligamentous complex among which the ligamentum flavum (LF) immediately posterior to the CSF-containing dural sac is of our interest. On T2-weighted images, the ALL and the PLL are seen as hypointense bands along the anterior and posterior edges of the vertebral column, while the LF is seen as a hypointense band extending along the posterior edge of the spinal canal (Figure 2).

**Figure 3:**
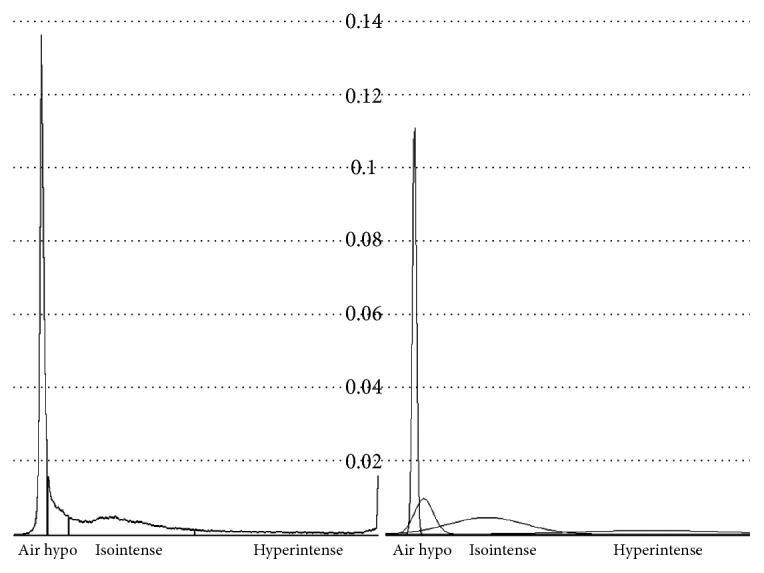
An example of classifying pixels on the histogram (left) after fitting with four Gaussian distributions (right). Horizontal dotted lines denote relative frequency.

We employ EM algorithm to cluster the pixels on the given midsagittal MR image according to their gray levels, or SIs. This method is widely used in processing brain MR images [14]. A Gaussian mixture model (GMM) is employed to fit the normalized histogram, as shown in Figure 3. This model assumes that the MR image consists of a number of distinct tissue types from which every pixel has been drawn. The intensities of pixels belonging to each of tissue type conform to a normal distribution, which can be described by a mean, a variance, and the number of pixels belonging to the distribution.

For human experts, visually classifying the pixels into hyper-, iso-, and hypointense ones is enough for making diagnoses. However, we frequently encountered problems in modeling the histogram using only three Gaussians because the hypointense pixels do not assume a perfect Gaussian distribution. Since there is no reason to assume that the hypointense ligament pixels share the same Gaussian distribution as the hypointense air pixels, we used two significantly overlapping Gaussians to fit the hypointense peak, increasing the total number to 4. Because the larger, narrower Gaussian mostly represents air pixels outside the body, we call it “air intense” to represent these very hypointense pixels. The other three Gaussians are called hyper-, iso-, and hypointense, respectively. Using EM, the number of Gaussian distributions is increased sequentially from one to four to fit the normalized histogram. Other details of the algorithm are implemented according to a textbook [15].

In spine images, isointense pixels are the most important as they usually represent the cord. The given gray level is classified isointense if the isointense Gaussian distribution contributes to the largest portion of that part of the histogram, as shown in Figure 3. Other pixels are classified similarly. Those with gray levels lower than the peak of the air intense pixels are automatically classified as such.

Although the EM algorithm always converges, sometimes the “isointense” Gaussian does not accurately represent true isointense pixels, that is, the cord. The SIs of the pixels can be affected, or “modulated,” by inhomogeneity of the radiofrequency field, placement of saturation bands, and adjustment by the MR operator [11]. On sagittal T2-weighted images, artifactual longitudinal thin linear hyperintensities are routinely seen, known as Gibbs artifact or truncation artifact [13]. Moreover, quantization of the SI into 256 gray levels also affects the EM process as the hyperintense Gaussian is often truncated if its mean is close to 256. As a result, relying on a fixed threshold to find the isointense pixels inevitably causes problems in some images.

From our pilot studies, we have found that the gray levels of the spinal cord pixels are mostly between 60 and 100. To cope with the errors associated with the EM algorithm, we checked the upper threshold of isointense pixels derived from EM. If it is larger than 127 or smaller than 64, adjustments are made for correct classification. If the threshold is larger than 127, it is recalculated according to the mean and standard deviation of the isointense Gaussian distribution. The resulting value is limited to the range between 127 and 159. On the other hand, if the upper threshold is smaller than 64, indicating three Gaussians assigned to the hypointense and air intense peaks, it is set to 128 without further recomputation.

Dynamic programming (DP) is a method of solving problems by combining the solutions to simpler subproblems [16]. It is typically applied to optimization problems to find a solution with the optimal value. “Programming” in this context refers to the method of tabulating the solutions of the subproblems. When the subproblems overlap, a DP algorithm solves each subproblem just once and then saves its answer in a table, thereby avoiding the work of recomputing it for many times.

In our application, we want to detect some anatomical structures on a midsagittal MR image using DP. We regard this image *I* as a large 320 × 320 checkerboard. The fitness or optimality of a given pixel at position (*y*, *z*), *f*_*y*,*z*_, can be defined locally using features derived from its gray level, *I*_*y*,*z*_, and from its neighboring pixels. Then, the optimal solution representing the structure to be detected is characterized as the best path *B*, which is composed of values {*b*_*z*_sup__, *b*_*z*_sup_+1_,…, *b*_*z*_inf__}, representing a series of points (*b*_*z*_sup__, *z*_sup_), (*b*_*z*_sup_+1_, *z*_sup_ + 1),…, (*b*_*z*_inf__, *z*_inf_) running from the uppermost row *z* = *z*_sup_ to the lowermost row *z* = *z*_inf_ within the region of interest. The path must be continuous, so one can only move from (*y*, *z*) to (*y* − 1, *z* + 1), (*y*, *z* + 1), or (*y* + 1, *z* + 1).

We define the cumulative fitness values of a given pixel at (*y*, *z*), *q*_*y*,*z*_, using *f*_*y*,*z*_ and values from its allowable predecessors, *q*_*y*−1,*z*−1_, *q*_*y*,*z*−1_, and *q*_*y*+1,*z*−1_. For the first row, *q*_*y*, *z*_sup__ is equal to *f*_*y*, *z*_sup__. When the pixel is out of the region of interest bounded by *y*_ant_ and *y*_post_, it is excluded from the best path. In our application, *z*_sup_ and *z*_inf_ are constants, while *y*_ant_ and *y*_post_ can be functions of *z* or constants.

(2) $$\begin{array}{c} q_{y,z}=\left\{\begin{array}{cc} 0 & \;if\;y<y_{ant}\;or\;y>y_{post}\\ f_{y,z} & \;if\;z=z_{sup}\\ max\left(q_{y-1,z-1,}q_{y,z-1},q_{y+1,z-1}\right)+f_{y,z} & otherwise. \end{array}\right. \end{array}$$

Starting from the uppermost row, the table storing of the cumulative fitness values is constructed. An auxiliary table *p*_*y*,*z*_ is also constructed to store the locations of the predecessors of a given point,

(3) $$\begin{array}{c} p_{y,z}=\left\{\begin{array}{c} -1\;if\;q_{y-1,z-1}>q_{y,z-1},\;q_{y-1,z-1}>q_{y+1,z-1}\\ 0\;if\;q_{y,z-1}>q_{y-1,z-1},\;q_{y,z-1}>q_{y+1,z+1}\\ +1\;if\;q_{y+1,z-1}>q_{y-1,z-1},\;q_{y+1,z-1}>q_{y,z-1}. \end{array}\right. \end{array}$$

If the maximum occurs in two or more predecessors, *p*_*y*,*z*_ is defined as 0 if *q*_*y*,*z*−1_ is the maximum and as −1 if *q*_*y*+1,*z*−1_ = *q*_*y*−1,*z*−1_ > *q*_*y*,*z*−1_.

To find *B*, we select the point with the largest cumulative fitness value in the lowermost row and then backtrack its predecessors using the auxiliary table until the first row is reached.

(4) $$\begin{array}{c} b_{z_{inf}}=\underset{y}{arg\;max}\left(q_{{y,z}_{\inf}}\right), \end{array}$$

(5) $$\begin{array}{c} b_{z-1}=b_{z}+p_{b_{z},z}\;for\;z=z_{inf}-1,\ldots ,z_{sup}. \end{array}$$

Let *f* ^*w*^ denote the fitness function used to compute the *w*th best path *B* ^*w*^. In the following sections, we use DP several times to find various anatomical structures represented by *B* ^1^, *B* ^2^,…, *B* ^8^ using *f* ^1^, *f* ^2^,…, *f* ^8^.

Connected to the brainstem, the spinal cord is the only isointense structure traversing the whole image vertically, as shown in Figure 2. The shape and size of the cord are limited by those of the bony spinal canal, which vary considerably. For patients with cervical spinal canal stenosis, those with a 7.1 mm canal depth, about 10 pixels deep in our images, were more likely to have CSM, whereas patients with a 10.8 mm canal were more likely to be nonmyelopathic [17]. Halving this value, we use 5 pixels as a reasonable estimate for the A-P diameter (depth) of the compressed cord.

In a given midsagittal image, the spinal cord can be detected by finding the longest isointense structure of sufficient depth. We define *f* ^1^ using classification results of 5 consecutive pixels in *y* direction,

(6) $$\begin{array}{c} f_{y,z}^{1}=\sum_{j=-2}^{2}u_{y+j,z},\;where\;u_{y,z}=\left\{\begin{array}{cc} 1 & if\;I_{y,z}\;is\;isointense\\ 0 & otherwise. \end{array}\right. \end{array}$$

The boundaries *z*_sup_, *z*_inf_, *y*_ant_, and *y*_post_ are set to 1, 320, 1, and, 320, respectively. Then, *B* ^1^ can be found using DP.

Since *B* ^1^ employs the results of the EM algorithm instead of the original SI, it can be fine-tuned using local SI homogeneity, as defined in *f* ^2^. Constants are added to ensure that *f* ^2^ has positive values everywhere. Similar adjustments are also used in (8) and (9).

(7) $$\begin{array}{c} f_{y,z}^{2}=\sum_{j=-3}^{2}\left(65536-{\left(I_{y+j+1,z}-I_{y+j,z}\right)}^{2}\right). \end{array}$$

**Figure 4:**
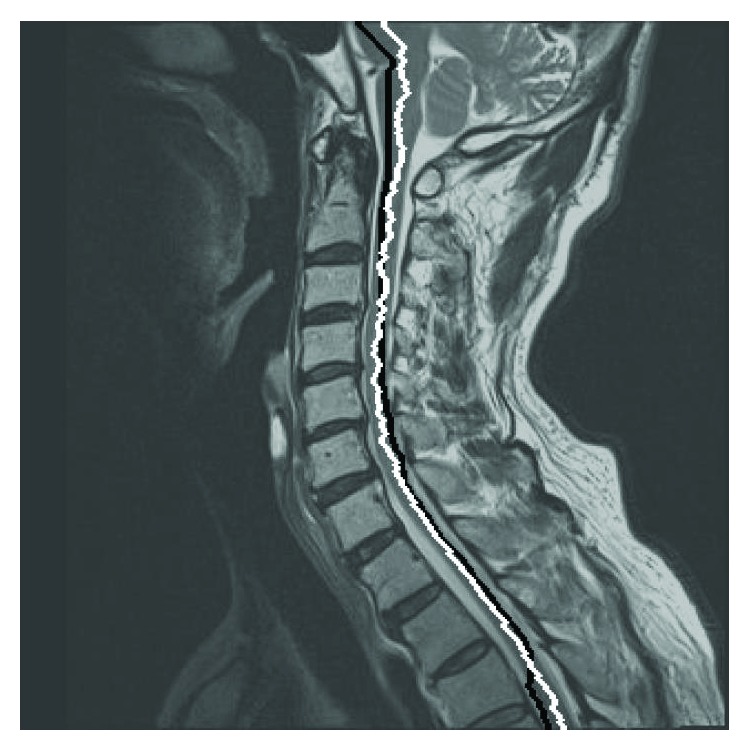
Spinal cord detection using dynamic programming. Near the black line indicating the best path containing the largest number of isointense pixels, the white line denoting the best path traversing the region having the most homogeneous signal intensity is detected.

Working around *B* ^1^, we compare 6 pairs of consecutive pixels to detect the best homogeneous isointense structure *B* ^2^, which is likely to be the cord, using DP. *z*_sup_,*z*_inf_, *y*_ant_, and *y*_post_ are set to 1, 320, *B* ^1^ − 40, and *B* ^1^ + 40, respectively. Although local zigzagging is common, *B* ^2^ is usually closer to the spinal cord along its course than *B* ^1^, as shown in Figure 4. Since *B* ^1^ and *B* ^2^ are not final segmentations, there is no need for them to be “centerlines” of the spinal cord.

Using *f* ^1^ and *f* ^2^, the exact location of the spinal cord can be detected on the sagittal image. If there is only one midsagittal image selected from the previous stage, the histogram along with pixel classification, *B* ^1^ and *B* ^2^ are saved and further processing is performed. If there are 2 candidate midsagittal images, we select one whose SI among pixels traversed by *B* ^2^ is more stable by comparing their SIs against the moving average derived from 31 neighboring pixels along the path, summed over the lower two-thirds of the image. Setting the range to 31 pixels, we hope to cover one VB and one intervertebral disk to decrease error associated with adjacent structures.

### 2.4. Ligament Detection Using Dynamic Programming

We define the range where the ligaments, namely ALL, PLL, and LF, may be detected, relative to the location of *B* ^2^, using measurements described in the literature [17]. The normal cervical spinal canal has an approximate depth of 15–20 mm, corresponding to 22–30 pixels in our images. The normal lower cervical VBs, including C3, C4, C5, C6, and C7, have approximate depths of 15–20 mm, corresponding to 22–30 pixels. They have approximate heights of 10–15 mm, corresponding to 14–22 pixels. Their normal areas on sagittal images range approximately from 300 to 600 pixels.

All ligaments appear hypointense on T2-weighted images (Table 1). Therefore, *B* ^3^ and *B* ^4^are defined in a straightforward fashion.

(8) $$\begin{array}{c} f_{y,z}^{3}=f_{y,z}^{4}={\left(256-I_{y,z}\right)}^{2}. \end{array}$$

Using DP, *B* ^3^ and *B* ^4^are found anterior and posterior to *B* ^2^. They represent PLL and LF, respectively. By setting *y*_ant_ and *y*_post_ relative to the location of the detected spinal cord, that is, *B* ^2^, we can ensure that no other hypointense structures will become the optimal solution erroneously. Based on normal spinal canal depth, the boundaries for *B* ^3^, *z*_sup_, *z*_inf_, *y*_ant_, and *y*_post_ are set to 1, 320, *B* ^2^ − 30, and *B* ^2^ − 1, respectively. Those for *B* ^4^ are set to 1, 320, *B* ^2^ + 1, and *B* ^2^ + 30.

It is more difficult to find ALL because the air-filled trachea is just anterior to it, separated by the thin isointense esophagus. We define *f* ^5^ in a slightly different way. In addition to the hypointense ligament and the cortical bone immediately behind it, we detect 16 isointense bone marrow pixels further posteriorly.

(9) $$\begin{array}{c} f_{y,z}^{5}={\left(256-I_{y,z}\right)}^{2}+\sum_{j=1}^{16}{I_{y+j,z}}^{2}. \end{array}$$

**Figure 5:**
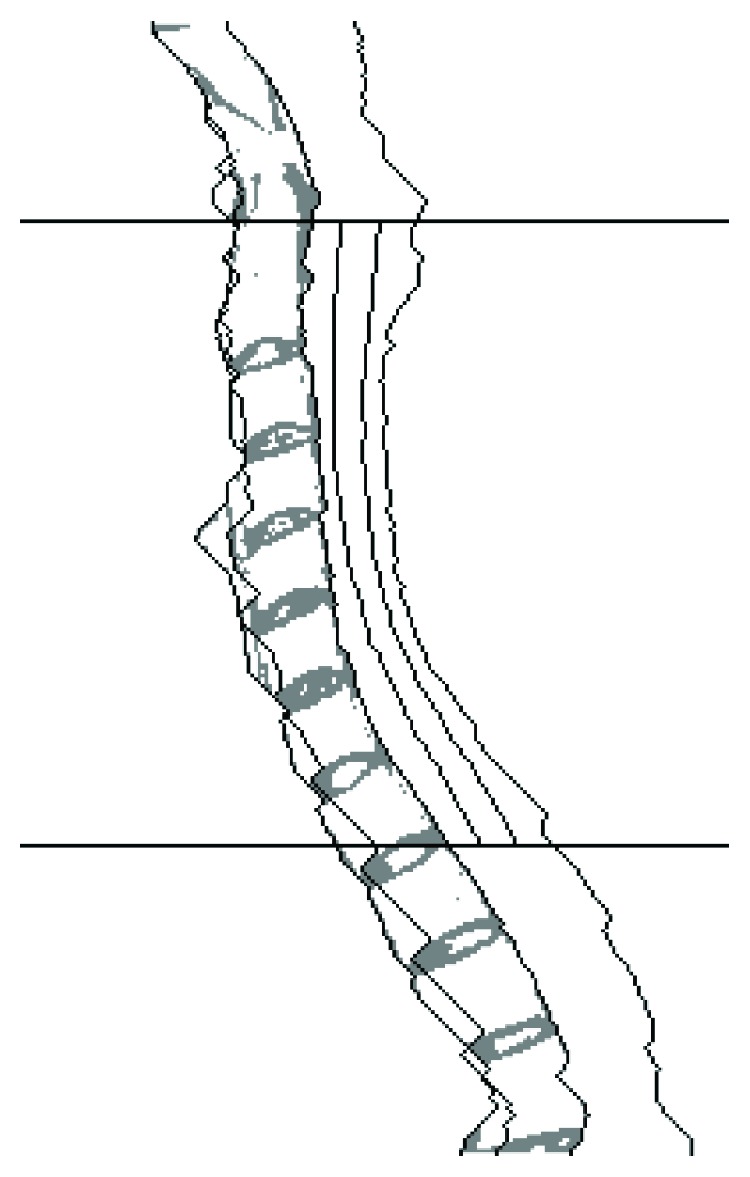
Detecting the edges of different structures using dynamic programming. From left to right: *B* ^5^, *B* ^6^, *B* ^3^, *B* ^7^, *B* ^8^, and *B* ^4^. The horizontal lines denote the superior and inferior edges of the spinal canal. The original MR image is shown in the left part of Figure 7.

Then, the approximate location of ALL, represented by *B* ^5^, is found using DP. Based on normal VB depth, the boundaries are set to 1, 320, *B* ^3^ − 60, and *B* ^3^ − 21 for *B* ^5^. After detecting *B* ^3^, *B* ^4^, and *B* ^5^, we can segment the key regions on the midsagittal image, namely the vertebral column and the spinal canal, as shown in Figure 5.

Although the vertebral column can be reliably detected using DP in most images, additional prevertebral soft tissue regions can be included, which may interfere with separation and detection of individual VBs. For atlas-dependent methods, the VBs and the spinal cord can be detected and labeled after image registration or other template-matching algorithm, usually after manual initialization [18]. Since our method is atlas-free and the only available information at this stage is spinal cord location, we must apply additional techniques to achieve the goal automatically.

### 2.5. Knowledge-Based Vertebral Body and Intervertebral Disk Detection and Labeling

The vertebral column, with its anterior and posterior edges defined by *B* ^5^ and *B* ^3^, contains the VBs and the intervertebral disks. The bony cortex of the VB, along with the annulus fibrosus and the end plates of the disks, is hypointense. Other structures of the vertebral column are isointense (Table 1). Therefore, we can construct a histogram of the vertebral column pixels to separate these two groups by finding the corresponding peaks and set the threshold, *t*_VB_, at the midpoint. This threshold is usually different from that defined to separate hypointense pixels from isointense ones in the EM process.

To facilitate separation of individual isointense bone marrow regions of the VBs, we need another fitness function *f* ^6^ to connect as many hypointense cortical bone and annulus pixels as possible. On the other hand, the posterior half of the VB regions must be retained for region growing algorithm to work.

(10) $$\begin{array}{c} f_{y,z}^{6}={\left(256-I_{y,z}\right)}^{2}+65536\sum_{j=1}^{20}v_{y+j,z},\text{ where }v_{y,z}=\left\{\begin{array}{cc} 1 & if\;I_{y,z}<t_{VB}\\ 0 & otherwise. \end{array}\right. \end{array}$$

The solution of the 6th DP process, *B* ^6^, usually lies between *B* ^5^ and *B* ^3^. It does not correspond to any specific structure but overlaps with *B* ^5^ at the anterior edges of the disks. Therefore, we call *B* ^6^ “truncated ALL.”

After thresholding all pixels between *B* ^6^ and *B* ^3^ using *t*_VB_, we employ exhaustive region growing to segment all isointense regions to find the VBs of the lower cervical spine. For each region, the location, height, depth, and area are calculated. Because some parts of the VB regions are outside the jagged *B* ^6^, we consider all regions larger than 150 pixels, that is, larger than half of normal VB size, being valid regions. Then, all regions are sorted according to their *z* coordinate in preparation for labeling.

The sizes and shapes for the lower cervical and upper thoracic VBs are relatively stable. In contrast, the C1 and C2 vertebrae assume complex shapes. Moreover, they are connected by other ligaments in addition to extensions of the ALL and the PLL, making their detection highly challenging. Despite such complexity, the odontoid process and the body of C2 vertebra usually appear as a connected region collectively, having a total height that averaged 30 mm in adults, about 1.5 times that of the lower cervical VB [19]. We use this knowledge to detect C2.

After detecting a large region followed by more than 5 valid VB regions inferiorly, the distance between the centers of the first and the second regions on the *z*-axis is checked. If it is smaller than 50 mm, or 70 pixels, these two regions are considered C2 and C3. Beginning from C3, all “regular” VB regions are labeled. The labeling process continues inferiorly for all VBs detected in the region growing process. The superior and inferior edges of the cervical spinal canal are approximated using the superior edge of the C2 region and the interior edge of the T1 region, respectively. An example is show in Figure 5. To avoid errors associated with vertebral regions at levels above C3 or below C7, we employ extrapolation on the *z*-axis from centers of the C3 and C7 VBs. The height of C2 is estimated as 1.5 times that of C3, while the height of T1 is estimated as that of C7. We intentionally include the T1 region so that the C7-T1 disk can be detected.

The SIs, sizes, and shapes of intervertebral disks change significantly with aging and various disease processes. Compared to detecting the VBs, detecting the disks is a much more difficult task. Therefore, we only attempt disk detection on images with successfully detected and labeled VBs. In late adulthood, the disks are dehydrated and the hyperintense region is minimal. However, the disk is still a region with heterogeneous SI, prohibiting improvement of segmentation accuracy using simple thresholding.

**Figure 6:**
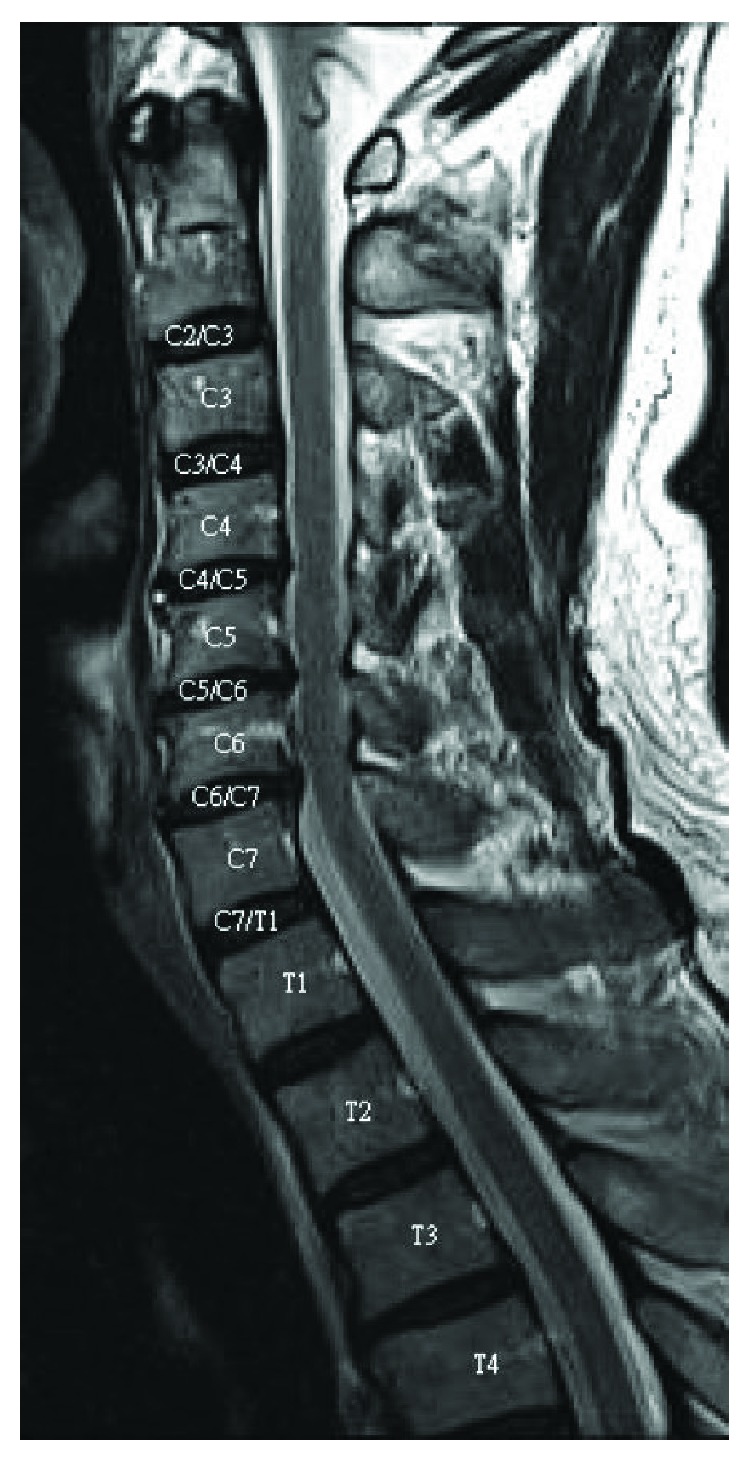
Vertebral body and intervertebral disk detection and labeling in a midsagittal MR image.

After removing the VB regions, the “void” regions within the vertebral column should be the disks. We use exhaustive region growing to detect the disk regions regardless of SI. Then, all regions larger than 100 pixels are considered disks and are labeled according to labels of the adjacent VBs. Since our method does not take SIs of the disk pixels into account, it is robust to disk pathologies, which commonly accompany CSM, as illustrated in Figure 6.

Although we have tested and tuned our algorithm in a pilot study, it still failed to identify pertinent structures on some images. Compared to the DP algorithm, the region growing algorithm used to detect VBs and disks is more sensitive to noisy SI. Therefore, we display the VB segmentation and labeling results to the user to allow corrections to be made. For images in which our algorithm fails to find or label the VBs, the superior and inferior edges of the cervical spinal canal can be designated by the user. At this stage, the user can also exclude images in which the complete cervical spinal canal does not exist or is not found by the algorithm from further processing.

### 2.6. Spinal Cord Border Detection Using a Compound Fitness Function

We perform cord segmentation after specifying the anterior, posterior, superior, and inferior edges of the spinal canal. Since there is no atlas for comparison, we have no information about how the SI of a given pixel is affected by various factors. To alleviate this problem, we apply median filtering to decrease noise [20]. For a given *z* within the spinal canal, we calculate the median gray level of the spinal cord, *c*_*z*_, using hypointense and isointense pixels within the range of *B*_*z*_^2^ − 5 to *B*_*z*_^2^ + 5 in *y* direction and *z* − 15 to *z* + 15 in *z* direction.

A compound fitness function is then constructed to maximize the contrast at the edge of the spinal cord, while keeping SIs within the spinal cord region as homogeneous as possible. Four terms representing similarity to the median cord gray level, heterogeneity between adjacent pixels, contrast between cord and noncord pixels, and penalty for passing through noncord pixels are incorporated into *f* ^7^,

(11) $$\begin{array}{c} f_{y,z}^{7}=f_{y,z}^{7s}+f_{y,z}^{7h}+f_{y,z}^{7c}+f_{y,z}^{7p}. \end{array}$$

The components are defined as follows. The weighting of each term is defined empirically using another training set of images.

(12) $$\begin{array}{c} f_{y,z}^{7s}=0.5{\left(I_{y-1,z}-c_{z}\right)}^{2}-3{\left(I_{y,z}-c_{z}\right)}^{2}-{\left(I_{y+1,z}-c_{z}\right)}^{2}-{\left(I_{y+2,z}-c_{z}\right)}^{2}-{\left(I_{y+3,z}-c_{z}\right)}^{2}, \end{array}$$

(13) $$\begin{array}{c} f_{y,z}^{7h}=-{\left(I_{y,z}-I_{y+1,z}\right)}^{2}-{\left(I_{y,z}-I_{y+2,z}\right)}^{2}-{\left(I_{y,z}-I_{y+3,z}\right)}^{2}, \end{array}$$

(14) $$\begin{array}{c} f_{y,z}^{7c}=0.5{\left(I_{y,z}-I_{y-1,z}\right)}^{2}, \end{array}$$

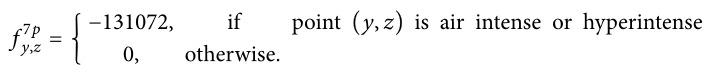
(15)


Similarly, *f* ^8^ is defined using the same components as *f* ^7^, with their constituent pixels in reverse order,

(16) $$\begin{array}{c} f_{y,z}^{8}=f_{y,z}^{8s}+f_{y,z}^{8h}+f_{y,z}^{8c}+f_{y,z}^{8p}. \end{array}$$

Using DP, the anterior and posterior edges of the spinal cord, *B* ^7^ and *B* ^8^, are detected. The region between them represents the spinal cord on the given T2-weighted midsagittal MR image.

The proposed segmentation method was validated against manual segmentation results. Similar to previous works, we used two measurements to validate the areas and edges resulted from the segmentation process [7]. The Jaccard index was used for quantifying the overlapping between cord regions defined by different observers.

(17) $$\begin{array}{c} J=\frac{TP}{TP+FP+FN}, \end{array}$$

where TP, FP, and FN denote the numbers of true-positive, false-positive, and false-negative pixels, respectively. It can be easily converted to Dice coefficient using the relationship *D* = 2*J*/(1 + *J*).

The Hausdorff distance, which is defined as the maximum distance between two curves, was used to quantify the distance between the anterior and posterior edges of the spinal cord. Three board-certified neurosurgeons performed spinal cord segmentation on the same images used for automatic segmentation. Interobserver agreements between them were calculated. Agreements between each human observer and the automatic method were also calculated. Then, the gold standard was determined using a voting process from three manual segmentations to assess the accuracy of the proposed method.

## 3. Results

A total of 84 eligible data sets from 84 patients were identified in the hospital database. All 1092 T2-weighted sagittal MR images were downloaded and processed. Our symmetry-based selection algorithm found 156 midsagittal images. These images were reviewed manually and were found to contain the odontoid process, which has an average width of 9 mm and is located near the iMSP [21]. Therefore, all of them were verified as midsagittal images.

Despite successful detection of midsagittal structures, 10 images from 5 data sets were excluded from further processing. In 4 data sets, no single MR image contains the complete cervical spinal canal due to excessive scoliosis, disqualifying the constraints of our algorithm. In one data set, the orientation of the spinal canal was significantly changed due to severe kyphosis related to thoracic wall deformity, prohibiting the DP algorithm to find appropriate solutions. Without the review process, these images would still fail VB detection and labeling and would be rejected for further processing instead of reporting erroneous automatic segmentation results.

From the remaining 79 data sets, a total of 146 images were selected as midsagittal images eligible for spinal cord detection. Among these 79 patients, there were 40 males and 39 females. Their ages ranged from 25 to 85 years, with a mean of 53.5 ± 12.0. After EM and two rounds of DP, the spinal cords were detected in all images. For each data set containing two midsagittal images, the one containing the more homogeneous spinal cord region was retained. After symmetry-based image selection and cord detection, the 7th image was selected in 45 (57%) of 79 data sets. The 6th and the 8th images were selected in 22 (28%) and 10 (13%) patients, respectively. The 5th image was selected in one and the 9th in another.

In 36 (46%) of the 79 images, the original threshold derived from the GMM was suitable for separating isointense pixels from hyperintense ones. For the remaining images, the thresholds were out of range. Automatic threshold adjustment using the isointense Gaussian was done in 43 (54%). In one image, the hyperintense pixels did not form an obvious peak on the histogram, resulting in absence of the pertinent Gaussian, and the upper threshold for isointense pixels was automatically set to 127.

Using DP, the PLLs were detected on all images without problem. The LFs were also detected on all images. After a manual review, small false-positive regions were noted in 6 cases and small false-negative regions in 2. These errors did not affect accuracy of cord segmentation. On the other hand, the results of ALL detection were less stable.

Prevertebral tissues were frequently considered part of the vertebral column.

**Figure 7:**
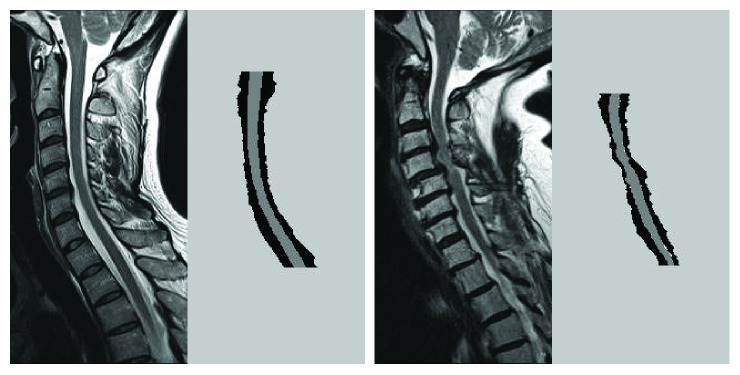
Segmentation results in nearly normal and severely degenerated cervical spines. The left half of each image is the original image containing the cord (gray) and its surrounding cerebrospinal fluid region (white). The right half of each image is the segmentation result. Erroneously classified pixels are shown in white. Components of the epidural space are also shown in some nonstenotic areas, but they are clinically irrelevant.

After truncating the vertebral column region using *B* ^6^ and subsequent region growing operations, 67 (85%) images had successful detection and labeling of the VBs. The labels were correct in 66 images. In one image, the congenitally fused C3-4 vertebral bodies were mistaken as C2 and the labels needed to be corrected. Manual designation for superior and inferior edges of the spinal canal was required in this image and in other 12 whose labeling was unsuccessful. Detection of the anterior and posterior edges of the spinal cord within cervical spinal canal was successful in all 79 images. Two examples are shown in Figure 7.

The heights of the spinal canal regions ranged from 150 to 231 pixels, with a mean of 187.8 ± 18.8. On average, males have longer canals than females (201.7 versus 173.5 pixels or 139 versus 119 mm) because they are taller. The number of spinal canal pixels ranged from 3000 to 5760, with a mean of 3943 ± 527. They account for only 4% of all pixels in the image. The number of manually segmented spinal cord pixels ranged from 1148 to 2473, with a mean of 1798 ± 250, and the number of automatically segmented spinal cord pixels ranged from 1155 to 2438, with a mean of 1803 ± 251. On average, the area of the spinal cord occupies about 46% of the spinal canal.

The mean gray levels of manually segmented cord pixels on the images ranged from 57.9 to 123.9, with a mean of 76.0 ± 9.3, while the standard deviations of these cord pixels ranged from 9.2 to 21.1, with a mean of 14.1 ± 2. Generally, the SIs of cord pixels decrease significantly as *z* increases. The mean correlation coefficient between the gray level and *z* was −0.57 ± 0.21 with a median of −0.63. In 65 of the 79 images, the correlation coefficients were lower than −0.4.

Compared to the gold standard, our algorithm had very good performance. The Jaccard indices ranged from 0.937 to 1, with a mean of 0.980 ± 0.014. Converted to the Dice coefficient, the range was 0.968 to 1, with a mean of 0.990 ± 0.007, better than that described in the previous study [7]. The Hausdorff distances between the automatically detected anterior spinal cord edge and the manually delineated one ranged from 0 to 3 pixels, about 0 to 2 mm, with a mean of 1.44 ± 0.67 pixels. The Hausdorff distances between the automatically detected posterior spinal cord edge and the manually delineated one ranged from 0 to 5.1 pixels, about 0 to 3.5 mm, with a mean of 1.47 ± 0.76 pixels.

Agreements between the results of spinal cord segmentation by our algorithm and by three human experts were shown in Tables 2 and 3. Both interobserver agreement measurements among three human experts were better than those between human and our algorithm.

## 4. Discussion

We have proposed an algorithm for automatic cervical spinal cord segmentation from original T2-weighted sagittal images. Our method is accurate and robust. All 156 midsagittal images selected from a total of 1092 were confirmed manually. We used EM on the histogram to find the upper and lower thresholds of isointense pixels. Although some adjustments were needed, our algorithm was able to find all spinal cords automatically, whose areas account for only 4% of all pixels in images having complete cervical spinal canals. Similar double threshold-based method was employed in other studies, but cropping regions of interest from the original images was needed before determining the threshold automatically [22].

Our method is completely atlas-free. The anatomical knowledge was built into the algorithm. Despite minimal human intervention, the results of our method were very accurate. The Dice indices and Hausdorff distances were better than those described in the previous studies [7]. In addition, our method is based on sagittal MR slice. These characteristics make our method complementary to current atlas-dependent methods based on axial images. Clinically useful metrics including cord compression and canal compromise described in [3] can be derived automatically on midsagittal MR images. Although similar knowledge of the spinal cord was incorporated in the methodology described previously [10], our emphasis other than anatomical structures, as detailed in Figure 2, has not been proposed.

When finding the MSP slices, we empirically define the difference metric of image pairs using the standard deviation of the corresponding pixels' gray level differences. However, the most commonly used tool for measuring image similarity is MI [14, 23]. We consider the slightly inferior performance of MI related to uncorrected radiofrequency field inhomogeneity and other artifacts as described in Section 2.3.

The stability of the EM algorithm is lower than we had expected. In addition to aforementioned sources of errors that also destabilize MI, adjustment of the histogram, or “windowing,” which helps radiologist reading the images, may also affect EM. After windowing, many hyperintense pixels stuck at the highest gray levels and their gray level distribution is no longer Gaussian. The adjusted threshold was at the allowed maximum in 6 of 43 images whose upper thresholds for isointense pixels were adjusted. In other images, the hyperintense pixels were also too heterogeneous to allow EM to fit a stable Gaussian for them. As a result, the adjusted threshold was at the allowed minimum in 32 images.

We use dynamic programming as the main tool used for detecting anatomical structures and their edges. When the cumulative fitness values *q*_*y*, *z*_inf__ are the same, there may be more than one optimal solution. Compared to the detection of PLL and the LF, detecting the ALL appeared much more difficult using DP. Variations of the prevertebral anatomy may play a role. Incorporating such knowledge may improve the segmentation accuracy for VBs and disks. It takes less than one minute to find the midsagittal slice and only seconds to segment the spinal cord because only two dimensions were used in the searching process. Although not directly comparable, the speed of our clinically oriented one-slice algorithm is considerably faster than the previous one using six dimensions [10].

We constructed two compound functions, *f* ^7^ and *f* ^8^, to detect the edges of the spinal cord. After considering various aspects affecting tissue contrast between the cord and its surrounding tissues, they seemed rather robust. Within the normal spinal canal, these functions accurately detect the interface between the isointense spinal cord and the hyperintense CSF region. When the CSF space disappears as compressed by the severely stenotic canal, the same functions can detect the interface between the isointense cord and the hypointense ligaments, as illustrated in Figure 7. However, when the width of the CSF region is minimized to one pixel, the partial volume effect may render it isointense, resulting in false-positive results.

Based on our algorithm, used alone or combined with others, one can develop a computer-aided diagnosis system capable of massive screening on cervical spine diseases, particularly CSM. During the review of the automatic spinal cord segmentation results, the human observers also evaluated the severity of canal stenosis. In most patients with moderate and severe stenosis, the changes in the anteroposterior diameter of the spinal cord are limited. On the other hand, changes in sizes of the CSF spaces are much more striking. If the cord diameter is used as the sole parameter measured in patients with CSM, disease severity may be underestimated. Therefore, some experts advocate correlating routine MR images to flexion-extension MR images if the diagnosis is in doubt [24].

There are several limitations to our algorithm that deserve mention. On the given midsagittal MR image, we used the spinal cord as the very first feature to be recognized and processed. Therefore, the algorithm cannot be applied to lower lumbar spinal levels, where the cauda equina composed of multiple nerves is only a neural structure within the canal. Although small regions of SI change within the spinal cord region caused by CSM did not affect its accuracy, our algorithm can fail when there are large cord lesions spanning long spinal levels. For the DP algorithm to detect the anatomical structures, a continuous spinal canal with the structures being aligned roughly and vertically is required. If the canal is disrupted by trauma, tumor, or other pathologies, modifications of our algorithm are required for it to work properly. Parameters of our algorithm must be tuned before application to other anatomical regions, such as thoracic and upper lumbar spines, as well as before application to other MRI scanners.

## 5. Conclusion

Automatic segmentation of the spinal cord and CSF in MR images remains a difficult task. We have presented an automatic method of spinal cord segmentation on sagittal T2-weighted images employing EM, DP, and region growing algorithms. Relevant anatomical knowledge is transformed into constraints in the algorithm enabling it to being atlas-free. Our method is accurate and robust and requires minimal human intervention. Used alone or combined with other methods, it lays foundation for computer-aided diagnosis of spinal diseases, particularly degenerative ones.

## Figures and Tables

**Table 1:** Signal intensities of different tissues on T2-weighted MR images. ALL: anterior longitudinal ligament; PLL: posterior longitudinal ligament; LF: ligamentum flavum.

Structure	Component	Signal intensity	Remark
Spinal cord		Isointense	Reference

Cerebrospinal fluid		Hyperintense	

Vertebral body	Cortical bone	Hypointense	
	Bone marrow	Isointense	May vary
	End plate	Hypointense	

Intervertebral disk	Annulus fibrosus	Hypointense	
	Nucleus pulposus	Hyperintense	Decreases with age

Ligaments (ALL, PLL, and LF)		Hypointense	

Air		Very hypointense	

**Table 2:** Interobserver agreements between the results of spinal cord segmentation by our algorithm and by three human experts, compared using Jaccard indices. Results in mean ± standard deviation and ranges in parentheses.

	Observer 1	Observer 2	Observer 3
Automatic segmentation	0.980 ± 0.015	0.979 ± 0.015	0.977 ± 0.015
	(0.922~1.000)	(0.937~1.000)	(0.939~1.000)
Observer 1		0.987 ± 0.010	0.987 ± 0.010
		(0.955~1.000)	(0.957~1.000)
Observer 2			0.989 ± 0.010
			(0.951~1.000)

**Table 3:** Interobserver agreements between the results of spinal cord edge detection by our algorithm and by three human experts, compared using Hausdorff distances. Results in mean ± standard deviation and ranges in parentheses.

	Anterior			Posterior		
	Observer 1	Observer 2	Observer 3	Observer 1	Observer 2	Observer 3
Automatic segmentation	1.51 ± 0.74	1.51 ± 0.66	1.54 ± 0.68	1.50 ± 0.76	1.44 ± 0.78	1.58 ± 0.79
	(0~3.61)	(0~3)	(0~3.16)	(0~4)	(0~5.83)	(0~5.10)
Observer 1		1.02 ± 0.43	1.01 ± 0.38		1.05 ± 0.55	1.05 ± 0.44
		(0~2.24)	(0~2)		(0~3.61)	(0~3)
Observer 2			0.97 ± 0.39			1.01 ± 0.57
			(0~2)			(0~3)
